# Supplementary material for: Prevalence and associated risk factors for hepatitis B and C viruses among refugee populations living in Mahama, Rwanda: A cross-sectional study
Source: PLoS One. 2021 Oct 11;16(10):e0257917. doi: 10.1371/journal.pone.0257917 (PMC8504757; doi:10.1371/journal.pone.0257917)
Supplement: S1 Appendix — (DOCX) [file pone.0257917.s001.docx]

**S1 Appendix. Association** **between demographic & clinical characteristics and having a viral load** ˃**20,000 IU/mL**

| **Characteristics** | **HBV DNA  ≤ 20,000 IU/mL** | **HBV DNA** ˃ **20,000 IU/mL** | **p-value** |
| --- | --- | --- | --- |
|  | N=556 | N=225 |  |
| **Sex** |  |  | 0.006 |
| Female | 242 (43.7%) | 73 (32.9%) |  |
| Male | 312 (56.3%) | 149 (67.1%) |  |
| **10-year age categories** |  |  | <0.001 |
| 15-24 | 93 (16.8%) | 69 (30.9%) |  |
| 25-34 | 212 (38.3%) | 80 (35.9%) |  |
| 35-44 | 144 (26.0%) | 47 (21.1%) |  |
| 45-54 | 66 (11.9%) | 18 ( 8.1%) |  |
| 55-64 | 26 ( 4.7%) | 7 ( 3.1%) |  |
| 65+ | 13 ( 2.3%) | 2 ( 0.9%) |  |
| **Self-reported co-morbidity** |  |  |  |
| Hepatitis B | 30 ( 5.7%) | 7 ( 3.2%) | 0.20 |
| Hepatitis C | 6 ( 1.1%) | 0 ( 0.0%) | 0.19 |
| Diabetes | 6 ( 1.1%) | 2 ( 0.9%) | 1.00 |
| Heart disease | 17 ( 3.2%) | 2 ( 0.9%) | 0.077 |
| Chronic renal failure | 8 ( 1.5%) | 2 ( 0.9%) | 0.73 |
| Cancer | 0 ( 0.0%) | 1 ( 0.5%) | 0.29 |
| **Household contact HBV RDT+** |  |  | 0.062 |
| No | 514 (92.9%) | 199 (88.8%) |  |
| Yes | 39 ( 7.1%) | 25 (11.2%) |  |
